# Radiotherapy in combination with vascular-targeted therapies

**DOI:** 10.2478/v10019-010-0025-9

**Published:** 2010-05-24

**Authors:** Eva Ciric, Gregor Sersa

**Affiliations:** Institute of Oncology Ljubljana, Ljubljana, Slovenia

**Keywords:** antiangiogenic agents, vascular-disrupting agents, radiotherapy

## Abstract

**Background:**

Given the critical role of tumor vasculature in tumor development, considerable efforts have been spent on developing therapeutic strategies targeting the tumor vascular network. A variety of agents have been developed, with two general approaches being pursued. Antiangiogenic agents (AAs) aim to interfere with the process of angiogenesis, preventing new tumor blood vessel formation. Vascular-disrupting agents (VDAs) target existing tumor vessels causing tumor ischemia and necrosis. Despite their great therapeutic potential, it has become clear that their greatest clinical utility may lie in combination with conventional anticancer therapies. Radiotherapy is a widely used treatment modality for cancer with its distinct therapeutic challenges. Thus, combining the two approaches seems reasonable.

**Conclusions:**

Strong biological rationale exist for combining vascular-targeted therapies with radiation. AAs and VDAs were shown to alter the tumor microenvironment in such a way as to enhance responses to radiation. The results of preclinical and early clinical studies have confirmed the therapeutic potential of this new treatment strategy in the clinical setting. However, concerns about increased normal tissue toxicity, have been raised.

## Introduction

Radiotherapy is an effective and widely used treatment modality for many tumors, with about half of all cancer patients undergoing radiation therapy as a part of their treatment.^1^ Although widely used, tumor radioresistance remains a major problem and a need exists to improve the cure rate by radiation therapy alone. As the patient population treated with radiotherapy is so enormous, enhancing the therapeutic outcome for even a relatively small proportion of these has the potential to translate to a highly significant clinical benefit. Combinations of cytotoxic chemotherapeutic agents with radiation have a synergistic effect on tumor response and are firmly established in clinical practice for a wide spectrum of tumors.^2^ In recent years, there has been increasing interest in combining vascular-targeted therapies with radiation.^3^ The enhanced antitumor efficacy of combined treatment may be explained by the alteration of the tumor microenvironment by vascular-targeted agents resulting in increased radiosensitivity of the tumor. However, the mechanisms of interaction between the two treatment modalities are complex and involve interactions between tumor stroma, the vasculature and the tumor cells themselves, which are not currently well understood. Therefore, the ideal way to use this potentially powerful combination for tumor cure has yet to be determined.

## Tumor angiogenesis

Angiogenesis is a critical step in tumor progression, as tumors are unable to grow beyond 2 mm^3^ without a vascular supply, due to lack of oxygen and nutrients.^4^ Formation of new blood vessels occurs from pre-existing vessels and allows the tumor to grow and expand rapidly.^5^ Tumors switch in their development to an angiogenic phenotype. The transition from dormant to the angiogenic state of the tumor is termed the “angiogenic switch” and is caused by a shift in the balance of anti- and pro-angiogenic factors.^6^ It is regulated by environmental factors and by genetic alterations that act to either up-regulate pro-angiogenic factors, such as vascular endothelial growth factor (VEGF) and basic fibroblast growth factor (bFGF) and/or down-regulate inhibitors of angiogenesis, such as angiostatin, endostatin, thrombospondin and interferons.^7^

The multistep process of tumor angiogenesis is characterized by degradation of the extracellular matrix, followed by proliferation and migration of the underlying endothelial cells into the tumor, with resultant vessel formation.^5^ The initial step in the process is activation of quiescent endothelial cells by binding of tumor-produced or stromal-produced growth factors to endothelial receptors. VEGF is a potent and specific growth factor that plays a pivotal role in endothelial cell activation.^8^ The main effects of VEGF are to increase vessel permeability and induce endothelial cell migration and proliferation, leading to the formation of endothelial sprouts, which then anastomose to form vascular loops and networks.^9^,^10^ VEGF also acts as a survival factor for endothelial cells by inhibiting apoptosis.^11^ It is therefore a pivotal driver of tumor angiogenesis, allowing tumor progression from in situ lesions to widespread disease, and providing the tumor with a route via which cells can get into the circulation and form distant metastases.^4^,^12^ VEGF is secreted by almost all solid tumors.^13^ Proliferating endothelial cells found in and around tumors produce multiple growth factors that not only promote endothelial cell growth but also tumor cell growth, invasion, and survival.^14^,^15^ Angiogenesis therefore provides both a perfusion effect and a paracrine effect for a growing tumor and tumor cells and endothelial cells can drive each other with resultant perpetuation and amplification of the malignant phenotype.^16^

Newly formed tumor blood vessels are distinct from those of normal tissue. They are markedly disordered, often dilated, tortuous and characterized by a relative lack of pericytes and other supporting cells, impaired blood flow and increased vascular permeability.^17^ Extravasation of macromolecules and pertinent development of high interstitial fluid pressure often results in vascular collapse, which leads to acidic and hypoxic areas heterogeneously distributed within the tumor mass.^18^,^19^ Hypoxia resulting from such functional vessel abnormalities is termed “acute” or “perfusion-limited”. The affected tumor cells are found much closer to blood vessels than would be expected from diffusion limitations and are exposed to oxygen concentrations that vary transiently between normal, anoxia and anywhere in-between. On the other hand, “chronic” or “diffusion-limited” hypoxia is found at an increased distance from blood vessels. In this type of hypoxia, individual perfused vessels are characterized by an oxygenation gradient surrounding them. Cells in this area exist at all possible oxygen concentrations ranging from anoxia at distant locations to normal values next to the vessels.^20^

## Hypoxia, angiogenesis and radioresistance

Hypoxia is an important stimulus for angiogenesis.^21^ Hypoxia inducible factor-1 (HIF-1) is a major mediator of the response to hypoxia. It is a transcriptional factor that regulates a number of processes, including VEGF transcription, apoptosis and cell cycle arrest.^22^,^23^ HIF-1 is regulated mainly by hypoxia, but it can also be activated in response to radiation.^24^ Both the hypoxic tumor microenvironment and external stresses such as ionizing radiation, lead to the up-regulation of many other pro-angiogenic factors, including VEGF, angiopoietin-2, nitric oxide synthase, platelet-derived growth factor (PDGF) and basic fibroblastic growth factors (bFGF).^25^,^26^ It has been shown that radiotherapy alone can potentiate angiogenic processes.^27^ Increased VEGF production in response to irradiation has been observed in various cancer cell lines.^28^ This is a part of the overall cellular response to stress and it is associated with the induction of a variety of transcription factors that can activate transcription of cytokines, growth factors, and cell cycle-related genes.

Hypoxia in tumors is strongly associated with radiation resistance as oxygen is required to chemically modify free-radical damage to the target DNA. When radiation is absorbed by the tissue, it creates reactive oxygen species that react with and damage cellular DNA, thus triggering cell death by apoptosis and/or necrosis. Cells irradiated in the presence of air are about three times more sensitive than cells irradiated under conditions of severe hypoxia.^29^ Pre-treatment measurements of tumor oxygenation have been shown to predict the response to radiotherapy and the likelihood of tumor recurrence, progression and metastatic disease in many human tumors.^30^ A more moderate hypoxia than is needed for maximum resistance to radiation has also been shown to have a negative impact on tumor control. This may be due to the fact that hypoxia influences a number of biological responses that affect tumor properties important for the treatment outcome, including angiogenesis.^20^ Different levels of hypoxia in a tumor thus provide the conditions for existence of viable cells that are not only radio-resistant but angiogenic as well.^31^,^32^

## Vascular-targeted therapies

The importance of targeting tumor vasculature development and function first became apparent in the 1970s through the seminal studies of Judah Folkman, who demonstrated that angiogenesis is crucial for the growth and survival of tumor cells. His findings suggest that both tumor cells and their supporting endothelial cells are potential targets for cell killing and should be considered when planning cancer treatment.^4^ Destroying the tumor vasculature deprives tumors of nutrients and oxygen necessary for their growth and should also inhibit metastatic spread, theoretically leading to tumor regression.

As a therapeutic group, vascular-targeting agents are unique as they have highly specific targets, while simultaneously having the potential to be effective against a broad range of tumor types. They are now divided into two classes; antiangiogenic agents (AAs), which inhibit the formation of new blood vessels, and vascular-disrupting agents (VDAs), which act against existing tumor vasculature. AAs are considered to be cytostatic in nature in contrast to VDAs, which are thought to be cytotoxic. Although there are differences between the two groups, including their administration schedules, individual agents might show both antiangiogenic and vascular-disrupting effects.^33^

### Antiangiogenic agents

AAs aim to prevent the growth of new blood vessels in tumors. One of the most widely studied targets for angiogenesis being explored clinically is VEGF and its receptors. VEGF is a ligand with a central role in signaling pathways controlling tumor blood vessel development and survival. The binding of VEGF ligands activates receptor tyro-sine kinases, designated VEGFR1, VEGFR2 and VEGFR3, which in turn activate a network of distinct downstream signaling pathways. Although the effects of VEGF receptors (VEGFR) signaling were initially thought to be specific for the vasculature, VEGF can also play a role in many other processes.^34^ VEGFR1 expression by colon cancer cells contributes to colon cancer cell motility and invasiveness but has little direct effect on proliferation of these cells. VEGFR2 expression by lung cancer cells may play a role in tumor cell survival after cytotoxic stress.^35^,^36^ Many different strategies for inhibiting VEGF activity have been evaluated, including the neutralization of the ligand or receptor by antibodies, blocking VEGFR signaling with tyrosine kinase inhibitors and even antiangiogenic gene therapy based on modulating the expression of VEGF pathway-related genes.^34^,^37^

Bevacizumab is a humanized monoclonal antibody that acts by binding and neutralizing VEGF. In a pivotal clinical trial conducted by Hurwtz *et al*., bevacizumab in combination with fluorouracil-based chemotherapy, significantly improved the overall survival for patients with metastatic color-ectal cancer over chemotherapy alone.^38^ Improved overall survival with combination therapy was also shown for patients with NSCLC and improved progression-free survival for patients with meta-static breast cancer and renal cell cancer was observed.^39^–^41^

Small molecule tyrosine kinase inhibitors (TKIs) present another class of antiangiogenic agents. They act by preventing activation of growth factor receptors, thus inhibiting downstream signaling pathways. They offer the theoretical advantage of being simultaneously active against receptors for different growth factors. Sunitinib, for example, targets VEGFRs, platelet-derived growth factor receptor (PDGFR) and c-kit and has shown significant efficacy in clinical trials for renal cancer.^42^

So far improvements in overall survival have only been seen in patients with colorectal and non-small cell lung cancers, when AAs were given in combination with chemotherapy. One possible reason why single-agent AAs ultimately fail is that there is up-regulation of other pro-angiogenic factors leading to angiogenesis and tumor resistance, hence the rationale for these drugs to be combined with chemotherapy or radiotherapy.^43^

### Vascular-disrupting agents

VDAs cause a rapid shutdown of perfusion in the established tumor vasculature, leading to tumor cell ischemia and secondary tumor cell death. These agents have the potential to destroy existing tumor masses and may be therefore particularly suitable for treating large tumors, which are typically resistant to conventional therapies.^33^

Two major classes of VDAs that selectively target tumor vessels are in clinical development; the ligand-directed VDAs and small molecule VDAs. Biological or ligand-directed VDAs work by using antibodies, peptides or growth factors which selectively bind to the endothelium. Coagulation and/or endothelial cell death is then achieved by coupling the vascular-targeting moiety with a toxin (*e.g*. ricin) or a pro-coagulant.^44^ Small molecule VDAs are at a much more advanced stage of clinical development than ligand-based therapies. These agents work by inducing vascular collapse, leading to extensive necrosis in tumors and include flavonoids and tubulin-depolymerizing/binding agents.^33^ Flavone acetic acid and its derivatives, particularly 5,6-dimethyl-xanthenone-4-acetic acid (DMXAA), have a complex mechanism of action and are believed to work by inducing the release of vasoactive agents and cytokines, such as tumor necrosis factor alpha (TNF-α), which leads to hemorrhagic necrosis.^45^ The tubulin-binding agents (*e.g*. combretastatin A-4 disodium phosphate) are believed to work by selective disruption of the cytoskeleton in proliferating endothelial cells in tumors. The subsequent change in endothelial cell shape leads to vessel blockage, thrombus formation, rapid reduction in tumor blood flow, and secondary tumor necrosis.^46^

Recently, electrochemotherapy has been recognized to have a vascular-disrupting effect besides a direct cytotoxic effect on tumor cells.^47^,^48^ Due to non-selective permeabilization of cells in the tumors exposed to electric pulses, endothelial cells also undergo apoptosis by uptake of bleomycin or cisplatin.^48^,^49^ This leads to permanent blood flow abrogation of the affected vessels leading to tumor hypoxia and necrosis, similar as in other vascular-disrupting agents.^50^ It has been estimated that the vascular-disrupting effect contributes 20–30% to the overall antitumor effectiveness of electrochemotherapy.^48^,^49^

The result of selective vascular destruction common to all of these strategies is extensive central tumor necrosis that leaves only a thin layer of viable cells at the tumor periphery. These cells are believed to obtain nutrients and oxygen from vessels of the surrounding normal tissue and their re-population may be the cause of treatment failure when VDAs are used in monotherapy, therefore combining VDAs with other standard treatment is an obvious option.^33^

## Combined treatments

As oxygen is crucial for maximal effectiveness of radiation, a logical concern when combining AAs and VDAs with radiation would be that compromising tumor vasculature by these agents would leave a tumor hypoxic and, thus, less radiosensitive. However, the mechanisms of interaction between the two treatment modalities have proved to be more complex and involve changes in the tumor microenvironment that may in fact result in an improved treatment outcome.^51^

### Radiotherapy and antiangiogenic agents

The understanding that tumor micro environmental factors, such as hypoxia, promote up-regulation of angiogenic and survival pathways leading to increased radioresistance, and that radiotherapy itself has pro-angiogenic effects, has prompted studies combining AAs with radiation.

**FIGURE 1:**
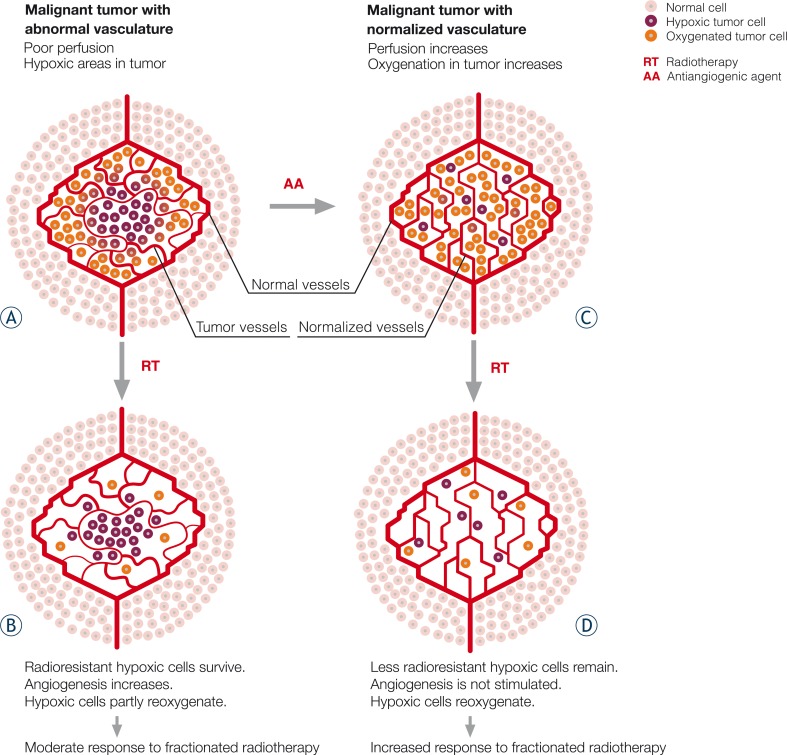
Theoretical model explaining the biological rationale for combining radiotherapy and AAs. A) Abnormal tumor vasculature largely composed of immature, disordered, often dilated and tortuous blood vessels is characterized by increased vascular permeability and impaired blood flow which leads to functional vessel abnormalities resulting in hypoxic areas in the tumor. B) After irradiation, oxygenated cells are destroyed, leaving behind the radioresistant hypoxic cells which release proangiogenic factors and further promote angiogenesis. During the time between radiation fractions hypoxic cells partly reoxygenate and further stimulate tumor repopulation, ultimately resulting in a moderate response to fractionated radiation. C) Pretreatment with AA destroys immature, inefficient tumor vessels and cause vessel reorganization thus increasing tumor perfusion and oxygenation. D) With irradiation many radiosensitive oxygenated cells are killed. The few remaining hypoxic cells reoxygenate, without angiogenesis being increased. The result is a less pronounced tumor repopulation and better overall response to fractionated radiation.

Teicher’s group was the first to show that AAs increase the tumor response when combined with single dose radiotherapy.^52^,^53^ A number of preclinical studies have since indicated that AAs can enhance the response to radiation (Table 1). The list of AAs evaluated in combination with radiation include non-specific antiangiogenic agent angiostatin, agents targeting the VEGF signaling pathway (anti-VEGF, anti-VEGFR antibodies and tyrosine kinase inhibitors), COX-2 inhibitors and epidermal growth factor receptor (EGFR) inhibitors which also target tumor cells. The antiangiogenic and antitumor effects have been reported to be additive as well as synergistic.^71^ Lee *et al.* conducted important animal experiments using an anti-VEGF antibody in combination with radiotherapy, resulting in synergistic antitumor effects. The anti-VEGF antibody decreased tumor interstitial fluid pressure and increased tumor perfusion, probably due to an observed reduction of tumor vascular density with vessel reorganization.^59^ In addition, AAs have been shown to increase oxygenation, thus increasing overall radiosensitivity. Jain tried to reconcile the paradoxical effects of AAs on oxygenation with the concept of “normalization” of the tumor vasculature.^72^ He postulated that rather than obliterating all tumor blood vessels, AAs destroyed only immature vessels, reduced vascular permeability and interstitial fluid pressure, and increased pericyte recruitment to stabilize intact vessels. Such normalization of tumor vasculature resulted in a more stable, organized vasculature, which could deliver oxygen and nutrients to the tumor more efficiently via well-functioning vessels, thereby decreasing hypoxia and hence radioresistance (Figure 1). However, continued antiangiogenic activity could cause vessel regression and impaired delivery leading to exacerbation of hypoxic conditions and radioresistance. Benefits of such a combination therapy may therefore be dependent upon a transient “normalization window” of opportunity when blood flow and tumor oxygenation are increased.^73^

Optimal timing for delivery of antiangiogenic therapy during the course of radiation to achieve the greatest enhancement of the radiation response, remains unknown and few studies have compared different sequences of radiation therapy and AAs.^74^ Recently, ZD6474 (vandetanib), a small molecule inhibitor of VEGFR2 with additional activity against EGFR, was combined with radiation therapy in the treatment of tumor xenografts. Two combination schedules were examined with vandetanib administered before each dose of radiation (concurrent schedule) or 30 minutes after the last dose of radiotherapy (sequential schedule). The growth delay induced using the concurrent schedule was greater than that induced by vandetanib or radiation treatment alone but the sequential schedule maximally delayed tumor growth. The authors demonstrated that a less pronounced response in the concurrent schedule was due to reduced tumor vascular perfusion caused by administration of vandetanib, which impaired re-oxygenation between radiation fractions, thereby decreasing radiosensitivity. In addition, the enhanced effect of vandetanib and radiotherapy in the sequential schedule could be explained by abrogation of VEGF-dependent survival signaling, which is supposed to have an important role in tumor recovery after irradiation.^65^

The enhancement of the effect of radiation therapy by antiangiogenic therapy may be also influenced by the tumor microenvironment. This was shown in a study by Lund *et al.* who treated mice with glioblastoma xenografts implanted into the thigh or intracranially with TNP-470 and/or radiation therapy.^55^ Significant enhancement of the tumor response to TNP-470 and radiation was seen in the thigh tumors, but no additive effect was observed in intracranial tumors. The authors proposed that differences in the capillary beds and microenvironment of the brain and the subcutaneous tissues of the thigh may have contributed to the differences in response.

### Radiotherapy and vascular-disrupting agents

**FIGURE 2:**
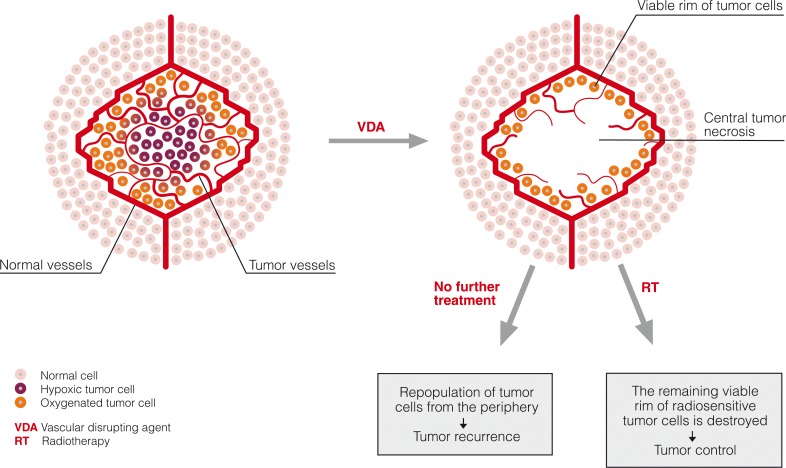
Schematic representation of the rationale for combining radiotherapy and VDAs. The result of VDA treatment is selective destruction of tumor vessels which causes extensive central tumor necrosis leaving only a thin layer of viable cells at the tumor periphery. These cells are believed to obtain nutrients and oxygen from vessels of the surrounding normal tissue and their repopulation may be the cause of treatment failure when VDAs are used in monotherapy. Combined treatment of VDA with radiotherapy may be more successful as radiation can destroy the viable tumor rim of well oxygenated and thus radiosensitive peripheral tumor cells remaining after the use of VDA.

The presence of a viable rim of tumor cells at the periphery after VDA treatment, as shown in pre-clinical studies, explains the modest tumor control seen in the single-agent phase I studies.^30^ It has been suggested that increased blood flow in the adjacent normal tissue, together with probable rapid up-regulation of angiogenic factors, such as VEGF, directly facilitates growth and expansion of the remaining rim of viable cells.^75^ These cells are believed to be well oxygenated and thus present an excellent target for conventional cytotoxic therapies. A logical rationale for combining VDAs with radiation would therefore be the interaction of the two treatments at the tumor microregional level; VDA reducing or eliminating the poorly oxygenated and hence radioresistant subpopulation of tumor cells and radiation killing the remaining well oxygenated peripheral cells (Figure 2). A number of pre-clinical studies performed on rodent tumor models over the past few years have reported enhanced tumor killing when VDAs were given in combination with radiotherapy (Table 2).

A study by Murata *et al.* showed the importance of scheduling.^83^ In his study for the murine CH3 tumors no improvement in local control was seen when combretastatin A-4 disodium phosphate was given 60 minutes before radiation compared to improved results when given concurrently or after radiotherapy. A likely explanation for this finding is that the vascular shutdown induced by the VDAs may have rendered some tumor cells hypoxic at the time of irradiation and that these cells later re-oxygenated and survived. It was suggested that blood flow needs to be re-established in the remaining viable tissue to obtain maximum radio-sensitization of the tumor. The greatest enhancement of the radiation response in fractionated dose regimens may be achieved when VDA is administered within a few hours after radiation. Under such conditions, antitumor effects may be greater than additive.^91^ An interesting animal study conducted by Siemann and Rojiani using the tubulin-binding agent *N-acetyl*-colchinol (ZD6126) and radiation showed that enhanced killing was more likely in larger tumors than in smaller ones.^89^ This observation may be explained by the fact that larger tumors are less radiosensitive due to increased hypoxic regions, which can be compensated by VDAs, whereas smaller tumors are more radiosensitive with fewer areas affected by VDAs.

Studies combining electrochemotherapy with tumor irradiation were also performed. The potentiation of the radiation response in experimental tumors was demonstrated with a single dose and fractionated radiation regime. A potentiating effect of 2.7 was observed with single dose irradiation and 4.6 with the fractionated regime.^92^–^95^ The effect of combined treatment was also demonstrated on tubal dedifferentiated papillary adenocarcinoma skin metastases.^96^ An enhanced radiation response with this treatment modality can be explained in part by radiosensitization of tumor cells that occurs in the process of electropermeabilization leading to increased uptake of radiosensitizing chemotherapeutic drugs, and in part by a vascular-disrupting effect, which is a result of electrochemotherapy as described in the previous section.

A therapeutic approach combining VDAs and radiotherapy may therefore be particularly suitable for treating larger tumors. The greatest antitumor effect may be achieved by administering VDA after radiation fractions. However, in order to determine the optimal treatment schedule in the course of fractionated radiation, further investigations are needed.

## Clinical trials on radiation and vascular-targeted therapies

The agents most widely explored in clinical trials are AAs targeting VEGF and its receptors. Of many explored in clinical trials, three have been approved for clinical use; two small molecule TKIs in monotherapy (sorafenib, sunitinib) for metastatic renal and hepatocellular carcinoma and an anti-VEGF monoclonal antibody (bevacizumab) in combination with chemotherapy for metastatic colorectal cancer, NSCLC and breast cancer.^97^–^101^ Today none of these agents is approved in combination with radiation therapy. However, several phase I and II clinical trials have been concluded and numerous are ongoing (Table 3). Many of the trials have showed a promising antitumor response. However, increased toxicity, such as fistula formation, wound healing problems and thrombosis, have been observed in some studies, especially when the VEGF inhibitor was combined with chemoradiotherapy protocols.^102^,^107^

VDAs are in a less advanced stage of clinical development, with only a few early trials concluded, mainly evaluating VDAs in monotherapy or chemotherapy combinations.^109^–^111^ Currently, the most widely explored VDA in clinical trials is combretastatin A-4 disodium phosphate, which has already been evaluated in several Phase I trials evaluating dosage schedules and toxicity, and has recently entered Phase II trials in combination with chemotherapy, radiation and radioisotopes.^51^

## Conclusion

Advances in the understanding of tumor biology have led to development of novel antitumor agents targeting tumor vasculature. Initial clinical trials testing these agents in monotherapy were somewhat disappointing and it has now become clear, that in most advanced malignancies, vasculature-targeting strategies will be most effective when used in combination with conventional anticancer therapies. Preclinical experiments on animal tumor models using different AAs and VDAs revealed possible mechanisms responsible for the synergistic antitumor effects of radiation and vascular-targeting strategies, based on AAs/VDAs altering the tumor microenvironment in such a way as to enhance responses to radiation therapy. The importance of treatment sequencing has been demonstrated in these preclinical studies.

Several early clinical trials combining AAs with radiation have showed the potential benefits of this treatment strategy in the clinical setting, warranting further investigations. However, the potential for higher rates of normal tissue toxicity has been documented, particularly in trials where AAs were combined with chemoradiotherapy. This indicates the need for careful design of future clinical trials with optimal radiotherapy planning and delivery in order to minimize damage to normal tissues. It might be prudent to first evaluate in early trials the combination of AAs/VDAs with radiotherapy alone. Further attention should be placed on the doses of AAs/VDAs, as currently there is little data suggesting that higher doses are necessarily better at enhancing the radiation response. Conventional strategies for monitoring anticancer therapies may not apply for vascular-targeted agents and clinical trials need to be designed not only to determine if the agents are safe and have evidence of efficacy, but also to validate both invasive and noninvasive surrogates of response. This will enable optimal treatment scheduling and, perhaps more importantly, selection of the patients and tumor types that will respond best to this new treatment strategy.

## Figures and Tables

**TABLE 1:** Preclinical combination trials with antiangiogenic agents and radiotherapy

**Antiangiogenic agent**	**Tumor model**	**Reference**
**TNP-470**	Lewis lung carcinoma	52, 53
	C3H mammary carcinoma	54
	U87 glioblastoma	55
**Angiostatin**	Lewis lung carcinoma	56
	D54 human glioblastoma	56
**Endostatin**	SQ-20B squamous cell carcinoma	57
**Anti-VEGF antibody**	Lewis lung carcinoma	58
	SQ-20B squamous cell carcinoma	58
	Seg-1 esophageal adenocarcinoma	58
	U87 glioblastoma	58, 59
	LS1747 colon adenocarcinoma	59
	Seg-1 esophageal adenocarcinoma	60
	U87 glioblastoma	59
**VEGFR-2 blockade**		
SU5416	GL261 murine glioblastoma	61
DC101	54A small cell lung cancer	62
	U87 glioblastoma	62
	MCa4 mammary carcinoma	63
	MCa35 mammary carcinoma	63
**VEGFR tyrosine kinase inhibitors**		
PTK787/ZK222584	SW480 human colon adenocarcinoma	64
ZD6474	CaLu-6 non-small cell lung cancer	65
	HT49 colorectal carcinoma	66
AZD2171	H460 non-small cell lung cancer	67
	CaLu-6 non-small cell lung cancer	68
	LoVo colorectal carcinoma	69
**Multi-kinase inhibitors**		
SU11248 (sunitinib)	Lewis lung carcinoma	69
	GL261 murine glioblastoma	69
SU6668	Lewis lung carcinoma	70
	GL261 murine carcinoma	70

**TABLE 2:** Preclinical combination trials with vascular-disrupting agents and radiotherapy

**Vascular disrupting agent**	**Tumor model**	**Reference**
**Tumor necrosis factor**	MCA-K mammary carcinoma	76
	MCA-K mammary carcinoma	77
**Flavone acetic acid**	C3H mammary carcinoma	78
**DMXAA**	RIF-1 fibrosarcoma	79
	MDAH-MCa4 mammary carcinoma	79
	C3H mammary carcinoma	80
	KHT sarcoma	80
**Combretastatin A-4 disodium phosphate**	KHT sarcoma	81
	Carcinoma NT	82
	C3H mammary carcinoma	83
	KHT sarcoma	83
	Kaposi’s sarcoma	84
	Rhabdomyosarcoma	85
**ZD6126**	C3H mammary carcinoma	86
	A549 NSCLC	87
	U87 glioblastoma	88
	KHT sarcoma	89
**MN-029**	KHT sarcoma	90

**TABLE 3:** Clinical trials of vascular-targeted agents in combination with chemoradiation/radiation therapy

**Vascular- targeted agent**	**Phase**	**Tumor**	**Treatment regiment**	**Reference**
**Bevacizumab (B)**	I	poor-prognosis head and neck cancer	chemoradiotherapy + B	102
	II	glioblastoma multiforme after surgery	temozolomide + radiotherapy + B → temozolomide + B	103
	II	locally advanced rectal cancer	standard preoperative chemoradiotherapy + B	104
	I/II	locally advanced inoperable colorectal cancer	chemoradiotherapy + B	105
	II	locally advanced inoperable pancreatic cancer	chemoradiotherapy + B → maintenance chemotherapy + B	106
		NSCLC	chemoradiotherapy + B	107
**Sunitinib (S)**	I	oligometastatic cancer	IGRT + S → maintenance S	108
**CA4P**		advanced NSCLC	palliative radiotherapy + CA4P	109

NSCLC = non-small cell lung cancer, IGRT = image-guided radiotherapy, CA4P = combretastatin A-4 disodium phosphate
